# A new generation needle- and adjuvant-free trivalent plague vaccine utilizing adenovirus-5 nanoparticle platform

**DOI:** 10.1038/s41541-020-00275-3

**Published:** 2021-01-29

**Authors:** Paul B. Kilgore, Jian Sha, Jourdan A. Andersson, Vladimir L. Motin, Ashok K. Chopra

**Affiliations:** 1 Department of Microbiology & Immunology, University of Texas Medical Branch, Galveston, TX USA; 2 Institute for Human Infections and Immunity, University of Texas Medical Branch, Galveston, TX USA; 3 Department of Pathology, University of Texas Medical Branch, Galveston, TX USA; 4 Sealy Institute for Vaccine Sciences, University of Texas Medical Branch, Galveston, TX USA; 5 Galveston National Laboratory, University of Texas Medical Branch, Galveston, TX USA

**Keywords:** Preclinical research, Bacteriology, Cellular immunity, Antibodies, DNA vaccines

## Abstract

A plague vaccine with a fusion cassette of YscF, F1, and LcrV encoding genes in an adenovirus-5 vector (rAd5-YFV) is evaluated for efficacy and immune responses in mice. Two doses of the vaccine provides 100% protection when administered intranasally against challenge with *Yersinia pestis* CO92 or its isogenic F1 mutant in short- or long- term immunization in pneumonic/bubonic plague models. The corresponding protection rates drop in rAd5-LcrV monovalent vaccinated mice in plague models. The rAd5-YFV vaccine induces superior humoral, mucosal and cell-mediated immunity, with clearance of the pathogen. Immunization of mice with rAd5-YFV followed by CO92 infection dampens proinflammatory cytokines and neutrophil chemoattractant production, while increasing Th1- and Th2-cytokine responses as well as macrophage/monocyte chemo-attractants when compared to the challenge control animals. This is a first study showing complete protection of mice from pneumonic/bubonic plague with a viral vector-based vaccine without the use of needles and the adjuvant.

## Introduction

*Yersinia pestis*, the causative agent of plague, is a Tier-1 select agent and a re-emerging human pathogen^1,2^. There have been three plague worldwide pandemics, which were responsible for over 200 million deaths^3^. The most recent large outbreak of plague (2017–2018) occurred in Madagascar which resulted in ~2400 cases and ~200 deaths^4^. Importantly, over 75% of the plague cases were pneumonic in this outbreak^5^. Traditionally, most cases of plague globally are bubonic in nature, which has a lower case-fatality rate and longer disease course than pneumonic plague^3^. Pneumonic plague, on the other hand, has a shorter disease course and an almost 100% fatality rate if not treated with appropriate antibiotics within 24 h of symptom onset, making it an urgent public health priority^6^. Indeed, Vallès et al. recently emphasized the importance of developing a clear roadmap on plague vaccines based on the current knowledge gaps^7^.

There are currently no Food and Drug Administration (FDA) approved vaccines to protect human population against plague^8,9^. However, FDA did provide “Orphan Drug” designation to the rF1-V vaccine (composed of recombinant capsular antigen F1 and a type 3 secretion system [T3SS] component [tip of the T3SS needle] and an effector, LcrV or V antigen) developed by DynPort Vaccine Company^10–12^. This vaccine has completed phase 2b clinical trials (https://clinicaltrials.gov/ct2/results?cond=plague&term=&cntry=&state=&city=&dist=). Plague subunit vaccines are generally administered in the host parenterally in conjunction with an adjuvant^13,14^. Likewise, a live-attenuated plague vaccine, *Y. pestis* EV76^15^, is used in humans outside the United States in the Former States of Soviet Union, Mongolia, and China. This vaccine strain, which lacks the pigmentation locus (*pgm*) needed to acquire iron from the host, is protective against both bubonic and pneumonic plague^16^. However, it is highly reactogenic and there are concerns about its safety in certain human populations such as those with hemochromatosis^17^.

There have been reports of naturally occurring F1-negative *Y. pestis* strains that can be as common as 10–16% in field sampling studies^18^. Importantly, F1-negative *Y. pestis* strains are still fully virulent^19^. Likewise, LcrV has hypervariable regions. There are at least five variants (clades) of LcrV based on the phylogenetic analysis of the conformational segment (amino acid [aa] residues 218–233) and the aa residue at position 255, from 43 *Y. pseudotuberculosis* strains^20^. Most importantly, LcrV variants from different clades when expressed from *Lactococcus lactis* did not offer cross-protection during infection in vivo models with *Y. pseudotuberculosis*^20^. Therefore, there is always a concern that rF1-V based subunit vaccines might not be fully protective against all *Y. pestis* strains, including the F1-negative and those which possess LcrV variants, that could cause an outbreak of plague^21^. Since *Y. pestis* has evolved from *Y. pseudotuberculosis*, plague-causing strains with LcrV variants are expected to exist in nature^22,23^.

We have previously developed a replication-defective human adenovirus-5 (Ad5) vector-based vaccine containing three *Y*. *pestis* antigens and included: YscF which constitutes needle of the T3SS, the capsule protein F1, and the T3SS component and effector LcrV^24^. The vaccine was designated as rAd5-YFV. Supplementary Fig. 1 depicted amino acid sequence of the rAd5-YFV construct. The details for generating rAd5-YFV trivalent- and rAd5-LcrV monovalent-vaccines, expression of the corresponding genes encoding LcrV and YFV (as determined by SDS-PAGE and western blot analysis using LcrV antibodies, as well as ELISA by coating plates with individual YscF, F1, and LcrV antigens), were provided in our earlier paper^24^.

The rAd5-YFV vaccine was initially tested either as a single dose alone (8.0 × 10^9^ virus particles [v.p] intranasally [i.n.] or intramuscularly [i.m.]) or followed by a booster of purified recombinant trivalent fusion antigen (rYFV) administered i.m. after 15 days of the first vaccine dose in a prime-boost strategy. One dose of the rAd5-YFV vaccine (delivered i.n.) offered significant protection (60%) in a pneumonic plague mouse model when challenged with ~100 LD_50_ of *Y. pestis* CO92. However, a rYFV protein boost was needed to achieve complete protection in both mouse as well as non-human primate models at much higher challenge doses of aerosolized *Y. pestis* CO92^24^.

Recently, the World Health Organization (WHO) has released preferred target product profile (TPP) of plague vaccines^25^. The recommendations included a needle-free vaccine that should not be administered in more than 2 doses, and the vaccine should generate long-lasting immune responses. In this study, we evaluated our rAd5-YFV vaccine delivered in 1 or 2 doses (21 days apart) by the i.n. route without a boost of the rYFV fusion protein. Subsequently, we compared rAd5-LcrV monovalent and rAd5-YFV trivalent vaccines in a short-term study (challenge on day 45 of the study, 24 days [~3 weeks] after the 2nd vaccination dose), and a long-term study (challenge on day 105 of the study, 85 days [~12 weeks] after the 2nd vaccination dose) for their efficacies. Robust humoral, mucosal, and cell-mediated immune responses were observed in mice immunized with the rAd5-YFV vaccine using a 2-dose vaccination regimen. The animals were fully protected from challenge with either parental *Y. pestis* CO92 or its isogenic F1 mutant in bubonic and pneumonic plague mouse models, with complete clearing of the pathogen. Furthermore, our data suggested that the T3SS needle structure protein YscF might be an important component in the rAd5-YFV vaccine formulation, especially against F1-negative *Y. pestis* CO92 challenge.

## Results

### A 2-dose rAd5-YFV trivalent needle-free vaccination regimen provides complete protection to mice against plague

We have previously shown that a single dose administration of the rAd5-YFV vaccine (8.0 × 10^9^ v.p.) by the i.n. route to mice provided complete protection against bubonic plague (subcutaneous, s.c., challenge), while 60% protection was achieved against pneumonic plague (i.n. challenge)^24^. Similar levels of protection were observed even when pre-existing immunity to Ad5 vector was induced in mice prior to vaccination^24^. Further, immunization of animals with the Ad5 vector alone did not influence humoral- and cell-mediated immune responses^24^.

**Fig. 1:**
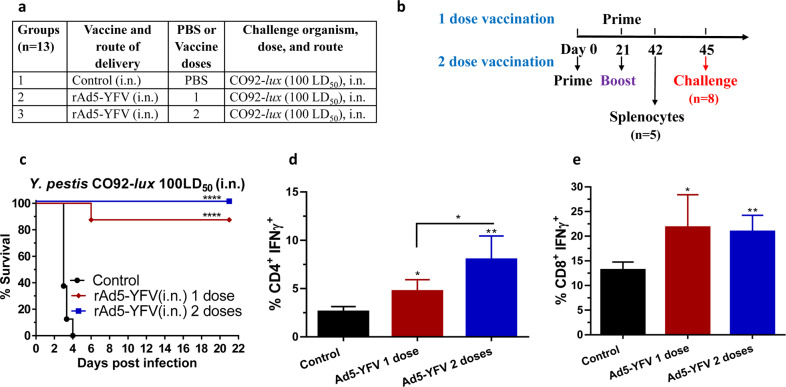
Protection and immune responses in mice immunized with 1 or 2 doses of the rAd5-YFV vaccine. Female Swiss–webster mice (*n* = 13/group) were immunized (i.n.) with either 1 or 2 doses (21 days apart) of rAd5-YFV vaccine (1.2 × 10^10^ v.p.), and animals administered PBS served as controls (**a**). Vaccination scheme is depicted in panel **b**. Twenty-four days after the final vaccination, eight mice from each group were i.n. challenged with 100 LD_50_ (5 × 10^4^ CFU/40 µL) of *Y. pestis* CO92-*lux*, and the survival of animals plotted (**c**). *P* values were calculated using Kaplan–Meier analysis with log-rank (Mantel–Cox) test for animal survival. Spleens were harvested from the five remaining unchallenged mice in each group and splenocytes stimulated with PMA and Ionomycin. Brefeldin A was added to prevent secretion of cytokines. Splenocytes were then stained for T-cell surface markers CD3, CD4, and CD8, followed by intracellular IFNγ staining. Percent of CD4^+^ IFNγ^+^ (**d**) and CD8^+^ IFNγ^+^ (**e**) T cells were analyzed by flow cytometry and the data expressed as the arithmetic means ± standard deviations. *P* values were calculated using a one-way ANOVA with Tukey post-hoc test to compare multiple groups or student *t*-test to compare 1 dose of rAd5-YFV vaccine to control in panel **e**. Asterisks above columns represent comparison to the control group, while horizontal bars represent differences between test groups. *P < 0.05, **P < 0.01, ****P < 0.0001. Two biological replicates were performed, and data plotted.

To improve efficacy of the rAd5-YFV vaccine against pneumonic plague, our first attempt was to increase the i.n. immunization dose from 8.0 × 10^9^ v.p. to 1.2 × 10^10^ v.p., and use vaccination regimen of either 1 dose or 2 doses in mice (Fig. 1a, b). As shown in Fig. 1c, animals immunized with 2 doses of the rAd5-YFV vaccine were completely protected (100%) compared to when animals received 1 dose of the vaccine (88% protection). In these experiments, animals were challenged ~3 weeks post last immunization dose (Fig. 1b). Further, while immune response analysis showed a similar percentage of CD8^+^ IFNγ^+^ T cells between these two groups of immunized mice (Fig. 1e), a significantly higher percentage of CD4^+^ IFNγ^+^ T-cell population was noted when animals were immunized with 2 doses of the vaccine over the 1-dose vaccination group (Fig. 1d). These data indicated that a better T-cell immune response was elicited with a 2-dose vaccination regimen.

To further evaluate the 2-dose immunization strategy with the rAd5-YFV vaccine, we included the F1-minus CO92 strain in addition to the parental CO92 for challenge studies. The capsular antigen F1 is one of the three immunogens in the rAd5-YFV vaccine, and F1-negative strains of *Y. pestis* exist in nature that are fully virulent. Thus, with the F1-minus CO92 challenge, the protection conferred by the rAd5-YFV vaccine is only dependent on antibodies to LcrV and YscF. Therefore, for comparison, we also included the monovalent vaccine rAd5-LcrV in the study to assess the role of YscF in the rAd5-YFV vaccine when challenging immunized mice with the F1-minus CO92 strain.

**Fig. 2:**
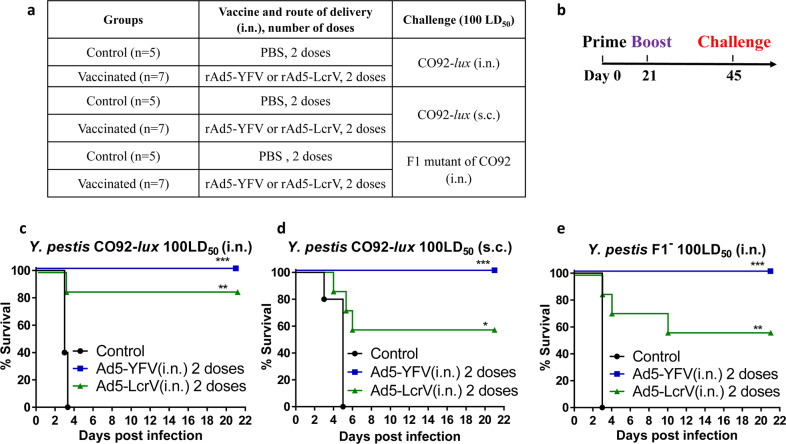
Animal protection conferred by immunization of mice with rAd5-YFV or rAd5-LcrV vaccines. Mice (*n* = 5–7/group) were immunized (i.n.) twice 21 days apart with either 1.2 × 10^10^ v.p. of rAd5-YFV or rAd5-LcrV vaccines, with mice receiving PBS served as controls (**a**). After 24 days post second immunization, animals were challenged with 100 LD_50_ of *Y. pestis* CO92-*lux* (**b**) by either i.n. route (**c**) or s.c. route (**d**) or by *Y. pestis* CO92 F1-negative strain via the i.n. route (**e**). The percent of animal survival was then plotted. *P* values were calculated using Kaplan–Meier analysis with log-rank (Mantel–Cox) test. Asterisks represent comparison to the control group. *P < 0.05, **P < 0.01, ***P < 0.001. Two biological replicates were performed, and data plotted.

As expected, both trivalent and monovalent vaccines provided protection to immunized mice albeit to varying degrees (Fig. 2). Various animal groups and the timeline for vaccination and challenge have been provided in Fig. 2a, b. When mice were challenged with the parental *Y. pestis* CO92 strain, we observed complete protection (100%) in the rAd5-YFV vaccine-immunized group, and an impressive 86% protection in the rAd5-LcrV vaccinated group, in a pneumonic plague mouse model (Fig. 2c). The efficacy of the rAd5-LcrV monovalent vaccine was decreased in a bubonic challenge model with only a 57% protection rate, while the rAd5-YFV trivalent vaccine provided 100% protection (Fig. 2d). Importantly, when the immunized mice were challenged with the F1-minus *Y. pestis* CO92 strain in a pneumonic plague model, the rAd5-YFV trivalent vaccine still conferred complete protection to mice, while the rAd5-LcrV monovalent vaccine provided only 57% protection (Fig. 2e).

### The rAd5-YFV trivalent vaccine elicits better humoral immune response than the rAd5-LcrV monovalent vaccine

**Fig. 3:**
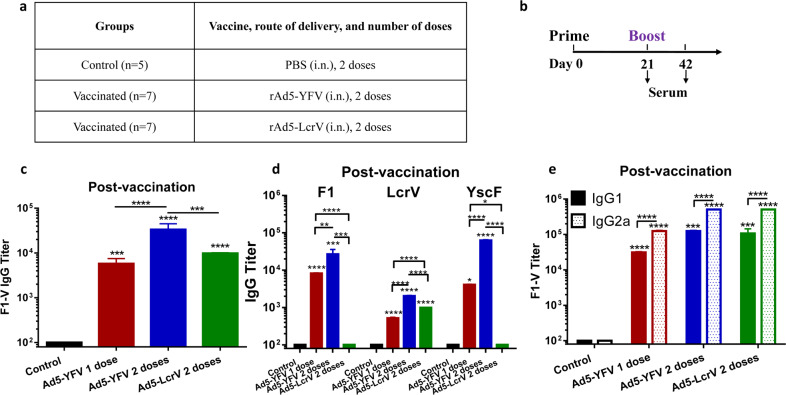
Antibody responses generated in mice immunized with rAd5-YFV or rAd5-LcrV vaccines. Mice (*n* = 5–7/group) were immunized (i.n.) twice 21 days apart with 1.2 × 10^10^ v.p. of rAd5-YFV or rAd5-LcrV vaccines, with animals that received PBS served as controls (**a**, **b**). Serum was collected on day 21 after the first dose as well as after the second dose of immunization (day 42) (**b**). The IgG antibody titers to rF1-V fusion protein (**c**) or to each individual antigen rF1, rLcrV, and rYscF (**d**), as well as the IgG1 and IgG2a isotype titers to rF1-V fusion protein (**e**), were measured by ELISA. The geometric mean of each sample ± standard deviations was used for data plotting. *P* values were calculated using a one-way or two-way ANOVA with Tukey’s post-hoc test to compare multiple groups or Student’s *t*-test to compare two groups. Asterisks above columns represent comparison to the control group, while horizontal bars represent differences between test groups. *P < 0.05, **P < 0.01, ***P < 0.001, ****P < 0.0001. Two biological replicates were performed, and data plotted.

We measured antibody titers to F1 and V in the sera of mice immunized with both the trivalent and monovalent vaccines using a most commonly used purified rF1-V fusion protein as the source of antigen. Various animal groups and the timeline for vaccination have been provided in Fig. 3a, b. A significant increase in anti-F1-V IgG antibody titers was noted after the second dose of rAd5-YFV vaccine. Importantly, the trivalent vaccine mounted significantly higher anti-F1-V IgG antibody titers compared to the rAd5-LcrV monovalent vaccine after 2 doses of the vaccination regimen (Fig. 3c).

To further profile the antibody titers, we measured IgG antibodies against the individual antigens (F1, LcrV, and YscF). We observed the same boosting effect on antibody production with significant increases in IgG titers against all three antigens after the second dose of the rAd5-YFV vaccine (Fig. 3d). As expected, no antibodies against F1 or YscF were detected in sera from mice vaccinated with the rAd5-LcrV monovalent vaccine. Further, antibody levels against LcrV were lower in mice vaccinated with 2 doses of the rAd5-LcrV monovalent vaccine than in mice that had been administered 2 doses of the rAd5-YFV trivalent vaccine (Fig. 3d). Interestingly, the antibody titer to YscF was the highest in the rAd5-YFV trivalent vaccine group of mice followed by antibody titers to F1 and then LcrV, indicating variation in immunogenicity and/or epitopes of the three antigens that were exposed in the rAd5-YFV vaccine (Fig. 3d). Some variations in the IgG titers in Fig. 3c, d could be reflective of antigens that were used to coat the microtiter plates and/or avidity of the antibodies to F1-V or individual F1, LcrV, and YscF antigens. Further, since antibody levels to F1 were higher than that of LcrV (Fig. 3d), it is expected that antibody titers to F1-V (Fig. 3c) would be similar to that of F1 titers observed in Fig. 3d.

Regarding isotypes of antibodies generated to F1-V, slight increases in both IgG1 and IgG2a titers were observed after the second dose of the rAd5-YFV trivalent vaccine (Fig. 3e). Both rAd5-YFV trivalent- and rAd5-LcrV monovalent-vaccines generated similar levels of IgG1 and IgG2a against F1-V (Fig. 3e) although the OD_450nm_ readings were higher for the rAd5-YFV trivalent vaccine than that of the rAd5-LcrV monovalent vaccine at a given dilution, without overall affecting the antibody titers. Further, significantly higher levels of IgG2a compared to that of IgG1 indicated a stronger Th1-biased immune response that was generated by both the vaccines (Fig. 3e).

### The rAd5-YFV trivalent vaccine induces better cell-mediated immune response compared to that of the rAd5-LcrV monovalent vaccine

**Fig. 4:**
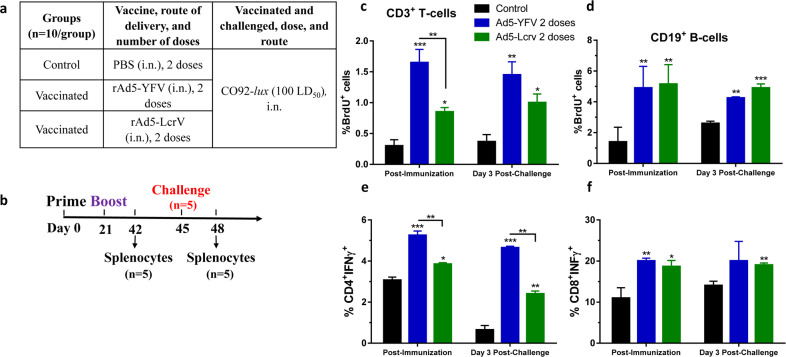
Cell proliferation and IFNγ production in mice immunized with either rAd5-YFV or the rAd5-LcrV vaccines. Mice (*n* = 10/group) were immunized (i.n.) twice 21 days apart with indicated vaccines as described in panel **a**. Spleens were harvested from mice (*n* = 5) either 21 days after the second vaccination dose or on day 3 post i.n. challenge with 100 LD_50_ of *Y. pestis* CO92-*lux* (**b**). For cell proliferation study after the second vaccination dose, splenocytes were stimulated with rF1-V (100 µg/ml) for 72 h at 37 °C, and then 10 µM BrdU was added during the last 18 h of incubation with the recombinant protein. For post-challenge timepoint, mice (*n* = 5) was i.n. challenged on day 24 after immunization (day 45 of the study) with *Y. pestis* CO92-*lux* (**b**), and 1 mg/100 µL BrdU was i.p. injected daily into mice for 3 days. On day 3 p.i., spleens were harvested after 1 h of last BrdU injection. The harvested splenocytes were then stained for T- and B-cell surface markers (CD3 and CD19) as well as for incorporated BrdU, and analyzed by flow cytometry. The percent of BrdU incorporation in CD3 (**c**) or CD19 (**d**) positive cells was plotted. For IFNγ studies (**e**, **f**), splenocytes were first stimulated with PMA and Ionomycin. Brefeldin A was then added to prevent secretion of cytokines. Cells were stained with T-cell surface markers CD3, CD4, and CD8 followed by intracellular IFNγ staining. Percentage of CD4^+^ IFNγ^+^ (**e**) and CD8^+^ IFNγ^+^ cells (**f**). *P* values were calculated using a one-way ANOVA with Tukey post-hoc test to compare multiple groups or student t-test to compare rAd5-LcrV CD8^+^IFNγ^+^ T cells to control during post-challenge timepoint. Asterisks above columns represent comparison to the control group, while horizontal bars represent differences between test groups. *P < 0.05, **P < 0.01, ***P < 0.001. Two biological replicates were performed, and data plotted.

We then compared cell-mediated immune responses generated by the trivalent versus the monovalent rAd5 vaccine. This was accomplished by measuring specific T- and B-cell proliferation in response to rF1-V stimulation (ex vivo) post-immunization as well as in response to *Y. pestis* infection (in vivo) on day 3 post-challenge of mice (Fig. 4a, b). The proliferation of immune cells was evaluated by measuring bromodeoxyuridine (BrdU) incorporation in dividing cells.

As shown in Fig. 4c, we observed higher T-cell proliferation in rAd5-YFV trivalent vaccine-immunized mice than that in the rAd5-LcrV monovalent vaccinated animals during both post-immunization and post-challenge. On the other hand, both rAd5-YFV trivalent- and rAd5-LcrV monovalent- vaccine-immunized mice had a similar level of B-cell proliferation, although their proliferation levels were significantly higher compared to their respective controls (Fig. 4d). Our earlier studies have convincingly shown that Ad5 vector- and rAd5-YFV-vaccinated mice did not contribute to any nonspecific cellular immune responses, including T-cell proliferation^24^. The cellular immune responses were only triggered after stimulating T cells with *Y. pestis* specific antigens.

To further investigate the T-cell response, interferon gamma (IFNγ) production was measured by flow cytometry both during post-immunization and at 3-day post-challenge with parental *Y. pestis* CO92 in mice (Fig. 4b). CD4^+^ T cells from the rAd5-YFV trivalent vaccine-immunized mice had a significantly higher percentage of IFNγ producing cells compared to the respective control, as well as to the rAd5-LcrV monovalent vaccine-immunized animals (Fig. 4e). There was no difference in percentage of CD8^+^ IFNγ^+^ T cells between rAd5-YFV trivalent and rAd5-LcrV monovalent vaccine-immunized mice, but their percentages were increased in both the vaccinated groups as compared to their respective control groups of mice (Fig. 4f). No nonspecific IFNγ production was noted when T cells from the Ad5 vector- and rAd5-YFV-vaccinated mice remained unpulsed, but significantly higher levels of IFNγ was produced when T cells were pulsed with rF1-V, based on ELISpot^24^.

We also measured cytokine and chemokine production in the splenocyte culture supernatants (Supplementary Fig. 2A–a and b). Compared to the respective controls (Supplementary Fig. 2B–D), the overall mouse cytokines and chemokines induced by either rAd5-YFV trivalent or rAd5-LcrV monovalent vaccine immunizations were similar in terms of their trends and levels. Significantly higher levels of proinflammatory cytokines IL-1α, IL-1β, and IL-6 were observed in control mice during infection. However, their levels remained low in either rAd5-YFV trivalent or rAd5-LcrV monovalent vaccinated animals both during immunization and post-challenge (Supplementary Fig. 2B). Significantly higher amounts of IFNγ, IL-4, and GM-CSF were observed in the rAd5-YFV trivalent vaccine-immunized mice when compared to animals vaccinated with the rAd5-LcrV monovalent vaccine in response to infection (Supplementary Fig. 2C, D). On the other hand, IL-13 and GM-CSF levels were substantially elevated in the rAd5-LcrV monovalent vaccinated mice (prior to infection) as compared to animals that were immunized with the rAd5-YFV trivalent vaccine (Supplementary Fig. 2C, D).

### The rAd5-YFV trivalent vaccination regimen induces a sustained protective immune response

**Fig. 5:**
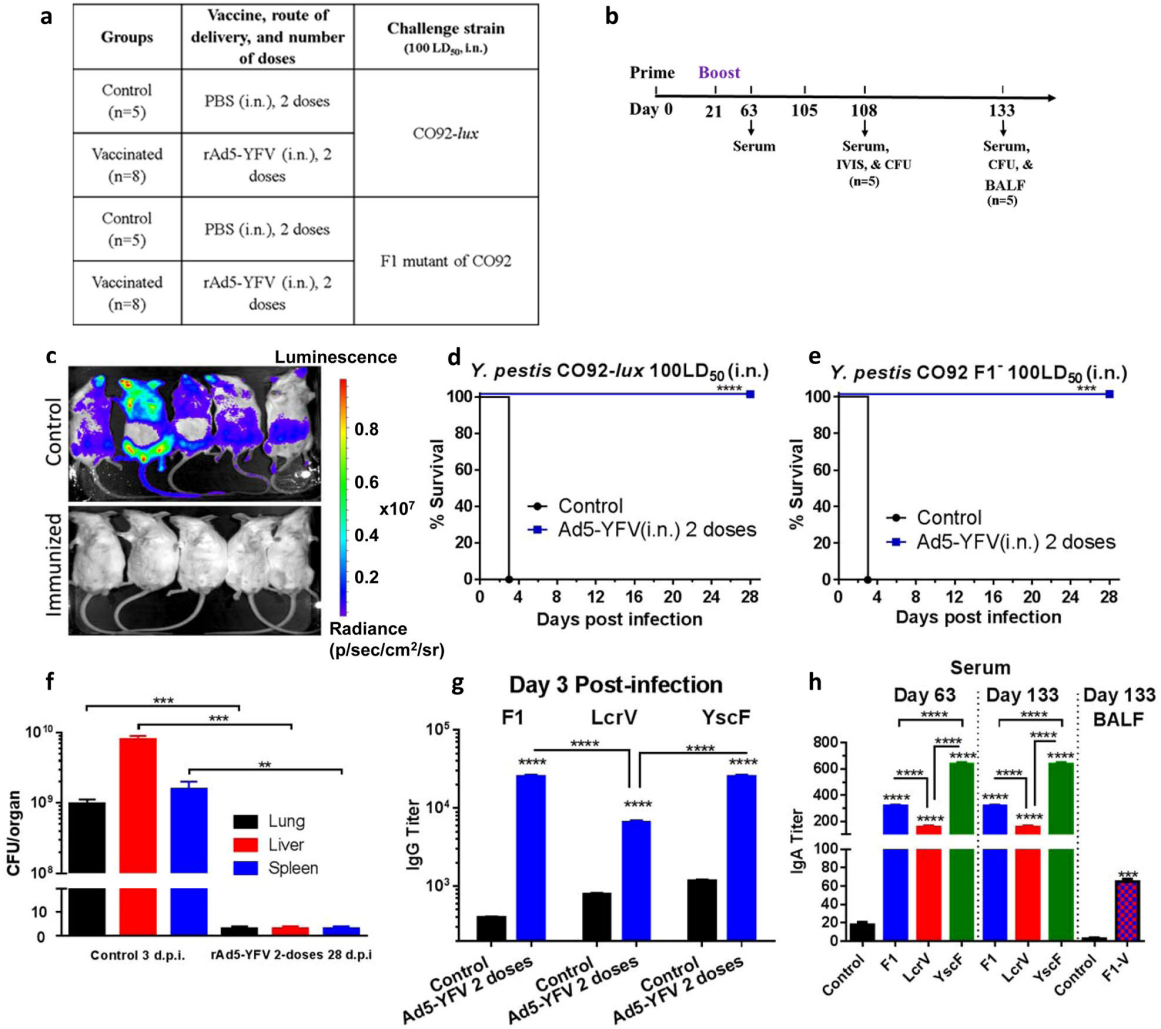
Long-term protection and antibody titers in mice immunized with 2 doses of the rAd5-YFV vaccine. Mice (*n* = 5–8/group) were immunized (i.n.) twice 21 days apart with 1.2×10^10^ v.p. of the rAd5-YFV vaccine, with animals receiving PBS served as controls (Fig. 5a). Eighty-four days (day 105 of study) after last vaccination, mice were i.n. challenged with 100 LD_50_ (5 × 10^4^ CFU/40 µL) of *Y. pestis* CO92-*lux* or *Y. pestis* CO92 F1-minus strain (Fig. 5b). The progression of infection in *Y. pestis* CO92-*lux* challenged mice was monitored by IVIS, and representative images of mice with a heat map of bacterial burden from lowest (violet) to highest (red) at day 3 p.i. are shown (Fig. 5c). The percent of animal survival was plotted (**d**) for the *Y. pestis* CO92-*lux* challenge or (**e**) for its F1-minus strain challenge. The actual bacterial loads in moribund control mice at day 3 p.i., and in the rAd5-YFV immunized mice at day 28 p.i. is displayed in panel **f**. On day 3 p.i., serum was collected from each group, and F1, LcrV, and YscF specific IgG titers were determined by ELISA (**g**). On days 63, and 133 (Fig. 5b), serum was examined for F1, LcrV, and YscF specific IgA titers by ELISA (Fig. 5h). At 28 days post-challenge, BALF was collected from mice in the *Y. pestis* CO92-*lux* challenged cohort and F1-V specific IgA titers were determined by ELISA (Fig. 5h). *P* values were calculated using Kaplan–Meier analysis with log-rank (Mantel–Cox) test for animal survival. One-way or two-way ANOVA with Tukey’s post-hoc test was used to compare multiple groups or Student’s *t*-test to compare two groups. Asterisks above columns represent comparison to the control group, while horizontal bars represent differences between test groups. *P < 0.05, **P < 0.01, ***P < 0.001, ****P < 0.0001. Two biological replicates were performed, and data plotted.

To assess the long-term protective immune responses, the immunized mice were challenged 85 days (12 weeks) after immunization (Fig. 5a, b), and the progression of infection monitored by whole body in vivo imaging. As shown in Fig. 5c at day 3 post-infection (p.i.), a strong bioluminescence detected in the challenged control mice was indicative of *Y. pestis* CO92-*lux* (with the luciferase gene) dissemination. However, the rAd5-YFV-immunized and challenged animals did not exhibit any bioluminescence at day 3 p.i., indicating clearing of the pathogen. All control mice in both *Y. pestis* CO92-*lux* and its F1-minus strain challenged groups went to moribund stage on day 3 p.i., while 100% of the rAd5-YFV-immunized ones survived from the challenges (Fig. 5d, e), correlating with nondetectable number of *Y. pestis* CO92-*lux* on day 3 p.i. (Fig. 5c, f). Furthermore, animal mortality was correlated with high numbers of plague bacilli present in multiple organs (lungs, liver, and spleen) of moribund control mice on day 3 p.i., while no *Y. pestis* was detected in the organs of rAd5-YFV-immunized and challenged mice neither on day 3 p.i. (Fig. 5c, based on bioluminescence) nor on day 28 p.i., based on standard bacterial plate counts (Fig. 5f).

We also observed that immunized mice maintained high IgG antibody titers to F1, LcrV, and YscF in sera on day 3 p.i. with *Y. pestis* CO92-*lux* (compare Fig. 3d and Fig. 5g). We also measured serum IgA levels against all three antigens on days 63 and 133 (Fig. 5b). As noted in Fig. 5h, higher IgA antibody titers were noted against F1 and YscF, with relatively lower IgA titers to LcrV, data which matched IgG antibody titers (Fig. 5g). After 28 days post-challenge (day 133), a significantly higher IgA titers to rF1-V were also detected in the bronchoalveolar lavage fluid (BALF) collected from the rAd5-YFV-immunized mice that survived the *Y. pestis* CO92-*lux* infection when compared to BALF collected from the uninfected control animals (Fig. 5h).

**Fig. 6:**
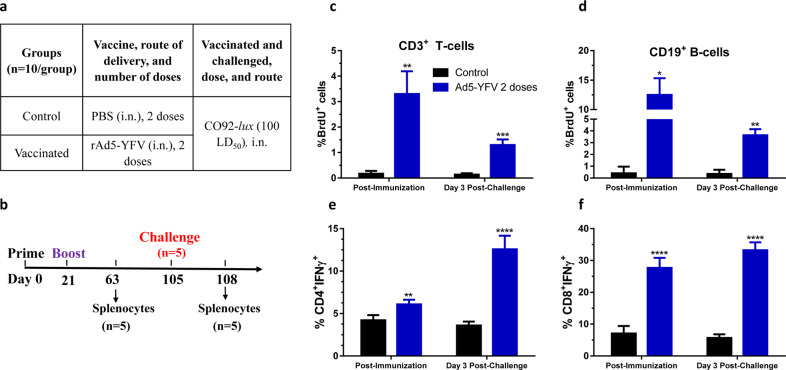
Cell proliferation and IFNγ production in mice immunized with rAd5-YFV vaccine during long-term study. Mice (*n* = 10/group) were immunized (i.n.) twice 21 days apart with rAd5-YFV vaccine (**a**). Forty-two days after the second vaccination (day 63 of the study), spleens were harvested from mice (*n* = 5) and also on day 3 post-challenge with *Y. pestis* CO92-*lux* (**b**). For cell proliferation study after the second vaccination, mouse splenocytes were stimulated with rF1-V and treated with BrdU for the post-vaccination timepoint (*n* = 5) as described in panel **b**. For the post-challenge timepoint, mice (*n* = 5) was i.n. challenged on day 85 after immunization (day 105 of the study) with *Y. pestis* CO92-*lux*, and i.p. injected with BrdU (**b**). On day 3 p.i., spleens were harvested and splenocytes were then stained for T- and B-cell surface markers (CD3 and CD19) as well as for incorporated BrdU, and analyzed by flow cytometry. The percent of BrdU incorporation in CD3 (**c**) or CD19 (**d**) positive cells was plotted. For IFNγ studies (**e**, **f**), splenocytes were stimulated with PMA, Ionomycin, and Brefeldin A. Cells were then stained with T-cell surface markers CD3, CD4, and CD8 followed by intracellular IFNγ staining. Percentages of CD4^+^ IFNγ^+^ (**e**) and CD8^+^ IFNγ^+^ cells (**f**) were shown. Student’s *t*-test was used to determine statistical significance between T-cell population from control and rAd5-YFV vaccinated groups. Asterisks above columns represent comparison to the control group. *P < 0.05, **P < 0.01, ***P < 0.001, ****P < 0.0001. Two biological replicates were performed, and data plotted.

We also observed that the rAd5-YFV immunized mice (Fig. 6a, b) had significantly higher levels of proliferating T cells (Fig. 6c) and B cells (Fig. 6d) post-immunization as well as on day 3 post-challenge with *Y. pestis* CO92 as compared to their appropriate controls (Fig. 6a). Similarly, the rAd5-YFV vaccinated mice had higher percentages of CD4^+^ IFNγ^+^ T cells (Fig. 6e) and CD8^+^ IFNγ^+^ T cells (Fig. 6f) compared to their respective controls (Fig. 6a) both at post-immunization and post-challenge of vaccinated mice.

### The rAd5-YFV trivalent vaccination regimen elicits strong cytokine and chemokine production in the long-term study

**Fig. 7:**
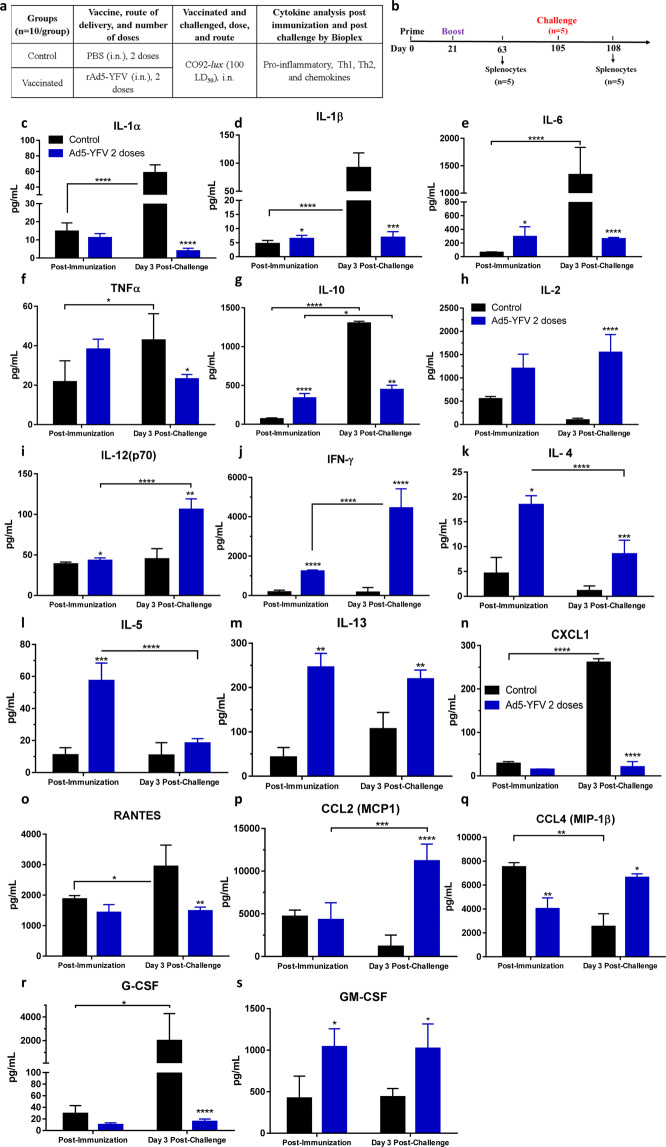
Splenocyte cytokine and chemokine profiles from the rAd5-YFV vaccine-immunized mice during a long-term study. Mice (*n* = 10/group) were immunized (i.n.) twice 21 days apart with 1.2 × 10^10^ v.p. of rAd5-YFV vaccine or rAd5-LcrV vaccine, with animals receiving PBS served as controls (Fig. 7a). Forty-two days after the second vaccination dose or on day 3 post-challenge, spleens were harvested from mice (*n* = 5/group/timepoint) and stimulated with purified rF1-V (100 µg/mL) for 3 days (**b**). The cytokines in the culture supernatants were analyzed by using Bioplex-23 assay and expressed as the arithmetic means ± standard deviations. The proinflammatory and anti-proinflammatory cytokines were listed in panel Fig. 7c–g, while the Th1 and Th2 cytokines were displayed in panel**s h**–**m**, and the chemokines as well as colony stimulating factors are presented in panels **n**–**s**. *P* values were calculated using a two-way ANOVA with Tukey post-hoc test to compare multiple timepoints or student *t*-test to compare two groups within the same timepoint. Asterisks above columns represent comparison to the control group, while horizontal bars represent differences between test groups. *P < 0.05, **P < 0.01, ***P < 0.001, ****P < 0.0001. Two biological replicates were performed, and data plotted.

We measured cytokine and chemokine production by splenocytes on day 63 post-immunization and day 3 post-challenge with parental *Y. pestis* CO92 strain (Fig. 7a, b). Based on our earlier studies^26^, we have shown that with the live-attenuated plague vaccines, significant levels of CD19^+^ CD38^+^ IgG memory B cells could be detected until day 63. However, by day 84, the percentage of these memory B cells were similar to that of the control. Similarly, percentage of CD4^+^ T cells producing IL-17 remained high until day 63, declining to that of control by day 84. Based on these data, we chose to examine cytokines/chemokines on day 63 post-immunization^26^.

In response to rAd5-YFV vaccination, the overall cytokines and chemokines in mice were either elevated or remained at compatible levels to those of control mice prior to infection (Fig. 7a, b). However, significant changes in cytokine and chemokine levels were observed during infection of immunized mice.

More specifically, the proinflammatory cytokines (IL-1α, IL-1β, IL-6, and TNF-α), as well as an anti-inflammatory cytokine IL-10, all were elevated in challenged control mice (Fig. 7c–g), but were generally unchanged in immunized mice in response to CO92 challenge (Fig. 7c–g). In contrast, cytokines related to a Th1-immune response [IL-2, IL-12(p70), and IFNγ] were significantly elevated in immunized mice, while these cytokines remained almost unchanged in control mice in response to an infection (Fig. 7h–j). On the other hand, the Th2-immune response cytokines (IL-4, IL-5, and IL-13) were induced in vaccinated mice compared to control animals. However, they either significantly subsided (IL-4 and IL-5) or maintained at a similar level (IL-13) in immunized mice in response to an infection with CO92 (Fig. 7k–m). It is important to note that vaccinated and challenged animals still had higher levels of IL-4 and IL-13 compared to challenged control mice (Fig. 7k, m).

For the chemokines and G-CSF (Fig. 7n–r), a similar level was observed in both control and immunized mice except for GM-CSF (Fig. 7s) which was significantly higher in immunized mice than in controls. However, in response to an infection, the levels of CXCL1, RANTES, and G-CSF were significantly higher in challenged control mice (Fig. 7n, o, r). In contrast, MCP1 and CCL4 were elevated in immunized and infected mice (Fig. 7p, q), while GM-CSF (Fig. 7s) remained at high levels in both immunized as well as in immunized and challenged mice.

## Discussion

In the aftermath of the 2017–2018 pneumonic plague outbreak in Madagascar, the WHO released a TPP outlining the desired characteristics of a successful plague vaccine^25^. These characteristics included: no more than 2-dose regimen, durability of protection, needle-free administration, universal coverage against all plague-causing strains, and stability during storage^25^. This outbreak of 2017–2018 seemed to mirror the third pandemic, which first established plague in Madagascar^7^, and served as a reminder to proactively react to prevent future plague outbreaks^7^.

Considering number of plague cases and deaths reported to WHO from 33 countries, including the US between 2013–2018 (a case-fatality rate of 17.5%), necessitates the development of new generation plague vaccines. Further, the recent finding that amoebae can serve as reservoirs for the plague bacterium is alarming^7^. Finally, the natural cellular immune response to pneumonic plague is not well understood, and to date, no phase III clinical trials have been conducted on any of the plague vaccines. Therefore, our study is timely to test a new generation plague vaccine in mice, and study in depth the immune responses.

As alluded to in our earlier paper^24^, we preferred to use Ad5 vector for developing plague vaccine because of its (i) well-characterized viral genome and the capability of integrating multiple genes; (ii) the use of Ad5 vector for gene therapeutic applications in humans; (iii) broad tropism infecting a variety of dividing and nondividing cells; (iv) ability of adenoviruses to effectively present transgene products to antigen presenting cells in vivo to promote rapid and robust humoral and cellular immune responses; (v) preferential induction of Th1-type immune responses, alleviating Th2-mediated eosinophil-related immunopathology; (vi) ability of adenoviruses to replicate to high titers in tissue culture cells; (vii) low biocontainment requirements for producing adenoviral vaccines, (viii) can be applied systemically as well as through mucosal surfaces, and adenoviruses are relative thermostable to facilitate their clinical use; (ix) inexpensive production, (x) no need for an adjuvant, and the vaccine can be delivered as aerosol mist stimulating potent and long-lasting humoral- and cell-mediated immune responses, and (xi) plug and play platform to develop multivalent/multicomponent vaccines.

Ad5 vector was also used in evaluating the efficacy of monovalent Ad5-F1 and Ad5-LcrV vaccines in a pneumonic plague mouse model using only the parental *Y. pestis* CO92 strain; however, the vaccines were administered by the i.m. route^27^. Further, we noted that while 1 dose of the rAd5-LcrV monovalent vaccine (8 × 10^9^ v.p.) provided only 20% protection to mice in a pneumonic plague mouse model when administered by the i.n. route, no protection was observed when delivered by the i.m. route at 90 LD_50_ of CO92, in spite of high antibody titers^24^. Our studies also showed that i.n. administration of the vaccine bypasses pre-existing antibodies to Ad5 vector to provide protection to animals^24^. Although Ad5 is the most prevalent serotype to which humans are exposed to, it induces minimal innate proinflammatory cytokine responses compared to other adenoviral serotypes used as vaccine vectors^28^.

Our 2-dose rAd5-YFV intranasal vaccination strategy essentially fulfills WHO’s TPP. The currently developed plague vaccines which underwent phase I and II clinical trials are based on F1 and V proteins mixed with alum^29^ or flagellin^13^ adjuvants. These vaccines induce a strong humoral response against both F1 and V antigens and have been shown to be efficacious in mouse and some non-human primate models of pneumonic plague. For example, consistent protection was observed in cynomolgus macaques; however, in African green monkeys, inconsistency in protection (0–75%) was noted in multiple studies^8,30,31^. The precise mechanism(s) for the lack of consistent protection has not been determined; however, humoral responses generated were similar for both non-human primate (NHP) models. It has been hypothesized that cell-mediated immune responses might be needed for complete protection in African green monkeys^8^, which are not adequately developed by the F1-V-based subunit vaccines.

While F1 and V are important antigens for plague vaccine development, there are some concerns that highlight the importance of including other plague immunogens to a potential universal plague vaccine. F1 capsule has been shown to be important for *Y. pestis* to resist phagocytosis during infection^32^. Strains of *Y. pestis* that lack F1, are still fully virulent in both pneumonic and bubonic models of disease albeit with a longer disease course seen during bubonic infection^19^. Bio-sampling studies have estimated that naturally occurring F1-negative *Y. pestis* strains could be as prevalent as 10–16%^18^.

Likewise, LcrV is a polymorphic protein which contains hypervariable regions within the COOH-terminal half where protective residues have been identified^20^. Studies have shown that vaccination with LcrV from different clades than the LcrV present in the challenge strain do not offer cross-protection during *Y. pseudotuberculosis* infection^20^. This scenario presents a situation where a potential outbreak strain of *Y. pestis* could avoid protection offered by a F1-V-based subunit vaccine.

In some earlier studies^30^, the efficacy of F1-V-based subunit vaccines against challenge with F1-negative *Y. pestis* strain at 11 weeks post-immunization, were reported. Likewise, the efficacy of a mutated *Y. pseudotuberculosis* strain expressing the gene encoding Caf1 when administered orally in mice against evoking bubonic and pneumonic plague was published^33^. In this study, we have evaluated efficacy of the rAd5-YFV vaccine (administered by the i.n. route) for the duration of 15 weeks of immunization, and also after challenge with F1-negative *Y. pestis* strain in a pneumonic plague model.

Our rAd5-YFV vaccine potentially could alleviate the issue of infection with either F1-negative *Y. pestis* strains or those harboring LcrV variants by the inclusion of a third antigen YscF, another component of the T3SS^34^. During parental *Y. pestis* CO92 pneumonic challenge, we observed slightly decreased protection (86%) by the monovalent rAd5-LcrV vaccine compared to 100% protection provided by the rAd5-YFV trivalent vaccine (Fig. 2c). The decrease in protection was even more pronounced (57% versus 100%) in a bubonic plague model (Fig. 2d). Furthermore, during F1-negative *Y. pestis* intranasal challenge, monovalent rAd5-LcrV vaccination only provided 57% protection, while the trivalent rAd5-YFV vaccination still conferred 100% protection (Fig. 2e). The higher level of protection conferred by rAd5-YFV immunization, especially against the F1-negative *Y. pestis*, could be contributed to the additional antigen YscF, as the F1 component in both vaccine (rAd5-LcrV and rAd5-YFV)-immunized mice would not have a role. However, we draw this conclusion with caution as LcrV epitopes might possibly be differentially exposed in rAd5-LcrV versus rAd5-YFV vaccines, which could afford better protection against infection with the latter vaccine. Such studies would be elaborated in the future.

In retrospect, we would prefer to have generated rAd5-FV vaccine as well. However, since we generated F1^−^ mutant of *Y. pestis* CO92^19^, we did not feel the necessity to develop rAd5-FV vaccine. We reasoned that infecting rAd5-LcrV-immunized animals with F1^−^ mutant of *Y. pestis* CO92 would provide the needed information for this study. We selected YscF as studies have shown that vaccination of mice with this T3SS needle structure protein provided protection to mice against subcutaneous injection with the encapsulated *Y. pestis* CO92^35^, and against an intravenously injected pigmentation locus-negative *Y. pestis* KIM strain^34^. In addition, we have also shown that serum from animals immunized with *Y. pestis* CO92 reacted strongly with YscF based on our proteomics studies (unpublished data). Therefore, we hypothesized that the protective antigen YscF could be used in combination with F1 and LcrV to formulate a more effective new generation trivalent rAd5-YFV vaccine.

The rAd5-YFV vaccine elicited stronger immune responses in mice compared to that of rAd5-LcrV vaccine. First, a much higher antibody titers were observed in mice immunized with the rAd5-YFV vaccine, and the higher antibodies were not only to the rF1-V fusion protein but also to each individual antigen (rF1, rLcrV, and rYscF) (Fig. 3c, d). We noted relatively higher IgG1 and IgG2a titers with both rAd5-YFV and rAd5-LcrV vaccines compared to IgG titers (Fig. 3). Biologically, it should not be the case; however, it is not uncommon and could be reflective of binding affinity and specificity of the secondary antibodies that were used. Indeed, higher levels of IgG subclasses over IgG titers have been reported in other studies as well^34,36–38^. Overall, both the trivalent and monovalent vaccines generated a stronger Th1 response based on IgG2a antibody titers (ratio of IgG2a/IgG1 ˃1)^39^. IgG1 and IgG2a have a differential role in animal protection against various infections^40,41^.

We observed significantly higher levels of T-cell proliferation in rAd5-YFV vaccine-immunized mice than those in rAd5-LcrV vaccinated animals when splenocytes were stimulated ex vivo with rF1-V antigen and labeled with BrdU (Fig. 4c). A similar trend was also seen when BrdU was injected during challenge of immunized mice and proliferation measured 72 h post-infection in vivo (Fig. 4c). Likewise, we also observed a significantly increased population of CD4^+^ IFNγ^+^ T cells both post-immunization as well as post-challenge when vaccination occurred with rAd5-YFV over rAd5-LcrV vaccine (Fig. 4e). Furthermore, substantially higher amounts of IFNγ and IL-4 were produced in the splenocyte supernatants of rAd5-YFV vaccine-immunized mice than those from rAd5-LcrV vaccinated animals in response to an infection (Supplementary Fig. 2C). These data further highlighted the advantage of rAd5-YFV over rAd5-LcrV as a plague vaccine candidate.

It has been reported that F1-V-based subunit vaccines adjuvanted with alum bias the immune response towards Th2 based on antibody isotyping^31,42^. In contrast, both rAd5-LcrV and rAd5-YFV vaccination generated strong Th1 and Th2-immune responses and were more biased towards Th1 based on the IgG2a/IgG1 antibody ratio, as well as the cytokine profile with overall higher magnitude of Th1 cytokines over Th2 cytokines (Fig. 3e and Supplementary Fig. 2C). A Th1-biased response is usually correlated with robust cell-mediated immunity that might be needed for complete protection in African green monkeys against pneumonic plague^43^.

A single dose of the rAd5-YFV vaccine provided impressive but incomplete protection (88%) in mice after challenge with parental CO92 (Fig. 1c). We noted a significant increase in anti-F1-V IgG antibody titers after the second dose of rAd5-YFV vaccine administration (Fig. 3). This boosting effect was also observed in the cell-mediated immune response, as increased levels of antigen specific CD4^+^ IFNγ^+^ T cells were observed in mice immunized with 2 doses of the rAd5-YFV vaccine compared to a single vaccine dose (Fig. 1d). While these data suggested that 2 doses of vaccine were needed to confer complete protection, 1 dose of the rAd5-YFV vaccine could be given during an outbreak emergency response scenario where there is not enough time to complete a full vaccination regimen as outlined in the TPP developed by the WHO^25^.

The TPP also stipulated that a viable vaccine should confer protection for at least 2 years in a reactive vaccination scenario and at least 5–10 years for an ideal preventative use scenario^25^. We detected high levels of antigen specific antibody titers in the rAd5-YFV vaccine-immunized mice on day 3 post-challenge, which was 88 days (~13 weeks) after last immunization (challenge day 108). These data suggested that 2 doses of the rAd5-YFV vaccine induced a sustained immune response in mice (Fig. 5g, h). It is difficult to correlate duration of protection in mouse models to length of protection that may be generated in humans due to a nonlinear relationship between life stages in mice and humans^44^. However, we did not observe a downward trend in antibody titers noted in other vaccine studies at similar timepoints when using 1- or 2 doses of the F1 + V based subunit vaccines^45,46^.

We also observed significant levels of F1, LcrV, and YscF specific IgA antibodies in the serum of vaccinated mice that survived 4 weeks of challenge compared to the control (Fig. 5h). While we did not measure IgA antibodies in the lungs of vaccinated mice prior to challenge, we noted higher levels of F1-V specific IgA in the BALF after 4 weeks of challenge (Fig. 5h). Antigen specific IgA antibodies, especially present in the airway, are important against respiratory infections, and immunization of animals *via* intranasal route is believed to facilitate the stimulation of mucosa immunity.

However, it is important to note that the role of IgA during pneumonic plague is not clear. In a study by Singh et al., IgA seemed dispensable in protecting mice against pneumonic plague^36^. In these studies, YopE-LcrV was produced from an attenuated strain of *Y. pseudotuberculosis*, and animals were immunized by the oral route. Our future studies will fully explore the role of IgA in protection using rAd5-YFV vaccine against pneumonic plague.

In addition to the induction of high levels of antibodies during the long-term study with the 2-dose vaccination regimen, we also observed significant T- and B-cell proliferation along with higher percentages of CD4^+^ IFNγ^+^ and CD8^+^ IFNγ^+^ cells in mice both after immunization and post-challenge (Fig. 6). These data confirmed that both humoral and cell-mediated immune responses generated were robust and long-lived, and these data were further supported by the splenocyte cytokine and chemokine profiles (Fig. 7) and the rapid clearance of the invaded pathogen (Fig. 5c, f).

In control animals, we noted a significant increase in proinflammatory cytokines (IL-1α, IL-1β, IL-6, and TNF-α), and chemokines (RANTES/CCL5 and CXCL1) coupled with the surge of G-CSF in response to infection (Fig. 7). This proinflammatory cytokine storm is a typical result of host neutrophil-macrophage interaction in recognition of invading pathogens^47^ and corresponds to the characteristic highly proinflammatory state of pneumonic plague (48–72 h p.i.), which leads to animal death if unresolved^48,49^.

The overwhelming proinflammatory cytokine storm is also the main cause of death for current COVID-19 patients, and the anti-inflammatory mediators, especially anti-RANTES, have been preferred for therapeutic intervention^43^. Importantly, this proinflammatory cytokine storm was prevented in rAd5-YFV vaccine-immunized mice after *Y. pestis* challenge, as all aforementioned cytokines and chemokines were largely unchanged when compared to their levels before infection (Fig. 7). The excessive level of IL-10 in infected control mice (Fig. 7) overall suppressed the immune response with a much-reduced level of Th1 and Th2 cytokines (Fig. 7). In contrast, the level of IL-10 remained low in immunized and challenged animals compared to that in control and infected mice (Fig. 7) with concomitant higher levels of Th1 and Th2 cytokines post-challenge (Fig. 7).

CXCL1 is an important chemoattractant for neutrophils. High levels of neutrophils are observed in uncontrolled plague infection, which correlate with the inflammatory cytokine storm. The latter results in immune-mediated damage to the lungs, contributing to the pathology seen during pneumonic plague^50^. On the other hand, we noted increased levels of monocyte/macrophage associated chemokines CCL2/MCP1 and CCL4/MIP1β in rAd5-YFV vaccine-immunized mice that were not seen in controls in response to CO92 infection (Fig. 7). Influx of activated monocytes/macrophages to the infection site is important for controlling infection, clearing dead cells, and reconstituting damaged tissue structures. Beyond chemotaxis, both CCL2 and CCL4 are also involved in modulating host immune response. It has been reported that CCL2 influences myeloid cell behavior, which leads to enhance host defense, cellular cleanup, allergic responses, as well as prime monocytes and macrophages in response to subsequent infections^51^.

Likewise, CCL4 plays a central role in the normal initiation of T-cell and humoral responses by recruiting CD4^+^CD25^+^ T-cell population^52^. In addition, we observed higher level of GM-CSF in rAd5-YFV vaccine-immunized mice than in controls (Fig. 7). Unlike G-CSF, which was increased in control mice in response to *Y. pestis* challenge to prolong neutrophil survival (Fig. 7), GM-CSF is a growth and differentiation factor for both granulocyte and macrophage populations^53^. Interestingly, GM-CSF has been reported to be involved indirectly in the induction of immunological tolerance and anti-inflammatory responses. More specifically, GM-CSF has been shown to facilitate T-cell-mediated tolerance by inducing “tolerogenic” dendritic cells (DCs)^53,54^. Therefore, the cytokines and chemokines elicited in rAd5-YFV vaccine-immunized mice obviously orchestrated a strong but controlled immune response to combat *Y. pestis* infection, while avoiding tissue damage, which correlated with the clearance of *Y. pestis* with no signs of disease, such as ruffled fur, lethargy, hunched posture, or lack of grooming. Our previous studies have shown that rAd5-YFV vaccinated NHPs did not show any histopathological lesions in any of the examined organs^24^.

In conclusion, rAd5-YFV vaccine with a 2-dose regimen induced humoral, mucosal, and cell-mediated immune responses that protected mice against pneumonic plague caused by parental *Y. pestis* CO92 and its F1-minus mutant. In the future, we will continue to investigate the importance of IgA antibodies and their role in protection from pneumonic plague as well as the potential for the rAd5-YFV vaccine to protect against *Y. pestis* strains with noncross-reactive LcrV variants. In addition, we will perform a more comprehensive comparison between rAd5-YFV and rAd5-LcrV vaccines (e.g., comparative expression of the *lcrV* gene, kinetics of the immune responses generated), and the individual contribution of YscF and F1 against protection.

## Methods

### Bacterial strains

Parental *Y. pestis* strain CO92 (also designated as CO92) is a fully virulent human pneumonic plague isolate acquired through BEI Resources (Manassas, VA). *Y. pestis* CO92-*lux* is a bioluminescent strain (with a luciferase reporter gene) created in the laboratory^55^. *Y pestis* CO92 F1 isogenic mutant was generated in our laboratory and demonstrated full virulence in pneumonic/bubonic plague mouse models^19^. All studies were performed in a Tier-1 select agent laboratory at UTMB within the Galveston National Laboratory (GNL), Galveston, TX.

### Animal studies

Six- to eight-week-old female Swiss–Webster mice were used in all studies. The experiments were performed in the animal biosafety level 3 (ABSL-3) facilities under an approved Institutional Animal Care and Use Committee protocol, UTMB, Galveston, Texas. Our earlier studies (unpublished) have shown that the gender, mouse species, as well as the age of mice did not affect the results.

### Immunization and antibody responses

Mice were immunized by the i.n. route with either one or two doses of the rAd5-YFV or rAd5-LcrV vaccines (1.2 × 10^10^ v.p./dose in a 20 µl volume per nostril, followed by a phosphate-buffered saline [PBS] wash, 10 µl volume). The two vaccine doses were delivered 21 days apart. Blood was collected via the retro-orbital route at the designated timepoints. The collected sera were filtered by using 0.1 µm filter cartridges (MilliporeSigma Life Science Center, Burlington, MA) and the sterility examined before subsequent procedures.

Antibody titers were determined by using indirect Enzyme-linked immunosorbent assay (ELISA). Briefly, ELISA plates were coated with 1 ng/µL of rF1-V (BEI Resources) or individual antigens rF1, rLcrV, or rYscF and allowed to adhere to the plates overnight at 4 °C^24^. Antigen coated plates were then blocked with 1% powered milk-PBS solution for 1 h at room temperature. Two-fold serial dilutions of sera were added to the plates and incubated for 1 h at room temperature. After 3 times washes with a 0.05% Tween 20 in PBS solution, horseradish peroxidase (HRP)-conjugated goat anti-mouse IgG, IgG1, IgG2a, or IgA antibodies (Southern Biotech, Birmingham, AL) (1:8000 dilution) were then applied for 1 h at room temperature. After three times washes, the reaction in the plates was developed using 3,3’,5,5’-Tetramethylbenzidine (TMB) solution (Sigma–Aldrich, St. Louis, MO) (100 µL/well) for 5–15 min at room temperature. The reaction was stopped using 2 N H_2_SO_4_ (50 µL/well). Color development was read on a Versamax tunable plate reader (Molecular Devices San Jose, CA) at 450 nm. Total IgG, its IgG1, and IgG2a isotype antibody titers, as well as IgA, were measured at least in 3 replicates.

### Animal challenge

For the short-term studies, animals were challenged 24 days after the last dose of the vaccine administration (day 45 of the study). For the long-term study, animals were challenged 85 days after the last dose of the vaccine delivery (day 105 of the study). Mice were anesthetized with isoflurane (to inhale) and challenged with 100 LD_50_ (1 LD_50_ corresponds to 50 or 500 colony forming units [CFU] for s.c. or i.n. challenge, respectively, to induce bubonic or pneumonic plague) by either parental *Y. pestis* CO92-*lux* or its F1-minus mutant^19^. Animals were observed for morbidity and mortality for 21–28 days. The progression of infection with *Y. pestis* CO92-*lux* was monitored by using an in vivo imaging system (IVIS) 200 bioluminescent and fluorescent whole body imaging system (Caliper Corp., Alameda, CA, USA) as previously described^55^. At designated timepoints (e.g., day 3 p.i. or the day of experiment termination), lungs, liver and spleen were harvested from animals to assess bacterial burden as we previously described^56^.

### Cell proliferation and cytokine production

To measure T- and B- cell proliferation, bromodeoxyuridine (BrdU), a thymidine analog, incorporation method was used. Briefly, spleens were aseptically removed from five animals of each group on either day 21 or day 42 after the last immunization dose, or day 3 p.i. with *Y. pestis-lux* CO92. Spleens were homogenized and passed through a 70 µm cell strainer to obtain single cell suspension in RPMI 1640 cell culture medium. Spleens from control mice served as a negative control. Splenocytes were then seeded into 24-well tissue culture plates at a density of 2.0 × 10^6^ cells/well and 4 wells/mouse/plate. For post-vaccination cell proliferation studies (ex vivo), the isolated splenocytes were first stimulated with rF1-V fusion protein (100 µg/ml, BEI Resources) for 72 h at 37 °C, and then BrdU (BD Bioscience, San Jose, CA) was added to a final concentration of 10 µM during the last 18 h of incubation with the recombinant protein to be incorporated into the splenocytes^57,58^.

Subsequently, the BrdU-labeled splenocytes were surface stained for T-cell (CD3e-APC; eBioscience) and B-cell (CD19-eFluor450, ThermoFisher Scientific, Grand Island, NY) markers after blocking with anti-mouse CD16/32 antibodies (BioLegend, San Diego, CA). Cells were then permeabilized and treated with DNase to expose BrdU epitopes followed by anti-BrdU-FITC and 7-AAD (7-amino-actinomycin D) staining by using BD Pharmingen FITC BrdU Flow Kit (San Jose, CA). The splenocytes were then subjected to flow cytometry, and data analyzed as we previously described^26^. The percent of BrdU positive cells in CD3- and CD19-positive populations were calculated using FACSDiva software. The gating strategy for flow cytometry is provided in Supplementary Fig. 3.

For in vivo cell proliferation measurements, mice were injected (i.p.) with 1 mg of BrdU at 24 h intervals during the first 3 days of infection with *Y. pestis-lux* CO92, and spleens were excised 1 h after the last BrdU injection^59^. Splenocytes were then isolated and stained for CD3 and CD19 surface markers as well as for intracellular staining of BrdU as described above.

To assess cytokine production, the splenocytes on the duplicate plates from both ex vivo and in vivo experiments (as described above) were simulated with rF1-V (100 µg/ml) for 72 h at 37 °C. Cell supernatants were then collected, and cytokines measured using Bio-Plex Pro Mouse Cytokine 23-plex Assay (Biorad Laboratories, Hercules, CA) following the manufacturer’s standard protocol.

### T-cell phenotypes

The splenocytes from duplicate plates from both ex vivo and in vivo experiments (as described above) were treated with ionomycin (750 ng/mL), PMA (phorbol 12-myristate 13-acetate) (50 ng/mL), and Brefeldin A (5 µg/mL) for 5 h at 37 °C in a 5% CO_2_ incubator. Splenocytes were then blocked with anti-mouse CD16/32 antibodies (BioLegend) followed by staining with Fixable Viability Dye eFluor™ 506 (eBioscience) and APC anti-mouse CD3e (eBioscience), PE/Dazzle 594 anti-mouse CD4 (BioLegend), FITC anti-mouse CD8 (BioLegend) for CD3, CD4, and CD8 T-cell surface markers, respectively. Cells were then permeabilized for intracellular staining with PerCP/Cy5.5 anti-mouse IFNγ (BioLegend) and analyzed by flow cytometry.

### Bronchoalveolar lavage and IgA production

Twenty-eight days after *Y. pestis* CO92-*lux* infection, bronchoalveolar lavage fluids (BALF) were obtained from the surviving animals as well as from uninfected control mice to assess IgA antibody titers. We obtained BALF following the protocol as previously described with slight modifications^60^. Briefly, the salivary glands were dissected to expose the trachea from euthanized mice (*n* = 5/group). A small incision was made on the ventral face of the trachea and a blunt 26 G needle was inserted into the trachea and secured by tying the trachea around the catheter using the floss placed underneath the trachea. An aliquot (600 µL) of PBS was loaded into a 1 mL syringe and attached to the needle. Next, the solution was injected and then aspirated three times in and out of the lungs before being collected. The anti-F1-V IgA antibody titers were determined by ELISA with the HRP-conjugated goat anti-mouse IgA antibodies (1:8000, Southern Biotech) as the source of secondary antibodies, followed by color development as described in an earlier section. Similarly, we examined F1, LcrV, and YscF specific IgA in the serum of mice at the designated times post-immunization and post-challenge.

### Statistical analysis

One-way or two-way analysis of variance (ANOVA) with Tukey’s post-hoc test or the Student’s *t*-test was used for data analysis. We used Kaplan–Meier with log-rank (Mantel–Cox) test for animal studies, and *P* values of ≤ 0.05 were considered significant for all the statistical tests used. The number of animals per group is described in each figure and two biological replicates were performed. All in vitro studies were performed in triplicates.

### Reporting summary

Further information on research design is available in the Nature Research Reporting Summary linked to this article.

## Supplementary information

Supplementary Information

Reporting Summary

## Data Availability

All data that this study is based upon are available from the corresponding authors upon request.
