# Supplementary material for: A new generation needle- and adjuvant-free trivalent plague vaccine utilizing adenovirus-5 nanoparticle platform
Source: NPJ Vaccines. 2021 Jan 29;6:21. doi: 10.1038/s41541-020-00275-3 (PMC7846801; doi:10.1038/s41541-020-00275-3)
Supplement: Supplementary file 1 — Supplementary Information [file 41541_2020_275_MOESM1_ESM.pdf]

Supplementary Figure 1.

```
MANFSGFTKGTDIADLDAVAQTLKKPADDANKAVNDSIAALKDKPDNPALLADLQHSINKWSVIYNINST
IVRSMKDLMQGILQKFPGGGGSGGGGSGGGGSADLTASTTATATLVEPARITLTYKEGAPITIMDNGNID
TELLVGTLTLGGYKTGTTSTSVNFTDAAGDPMYLTFTSQDGNNHQFTTKVIGKDSRDFDISPKVNGENLV
GDDVVLATGSQDFFVRSIGSKGGKLAAGKYTDAVTVTVSNQGGGGSGGGGSGGGGSMIRAYEQNPQHFIE
DLEKVRVEQLTGHGSSVLEELVQLVKDKNIDISIKYDPRKDSEVFANRVITDDIELLKKILAYFLPEDAI
LKGGHYDNQLQNGIKRVKEFLESSPNTQWELRAFMAVMHFSLTADRIDDDILKVIVDSMNHHGDARSKLR
EELAELTAELKIYSVIQAEINKHLSSSGTINIHDKSINLMDKNLYGYTDEEIFKASAEYKILEKMPQTTI
QVDGSEKKIVSIKDFLGSENKRTGALGNLKNSYSYNKDNNELSHFATTCSDKSRPLNDLVSQKTTQLSDI
TSRFNSAIEALNRFIQKYDSVMQRLLDDTSGK
```

Supplementary Figure 1. Schematic depicting the amino acid sequence of YFV fusion protein in Ad5 vector. YcsF (yellow), F1 (gray) and LcrV (cyan) were connected by a flexible linker of 3 GGGGS sequences (bold and red). The construction of rAd5-YFV and rAd5-LcrV has been detailed in our earlier paper[24].

Supplementary Figure 2A.

a

Groups (n=10/group)	Vaccine, route of delivery, and number of doses	Vaccinated and challenged, dose, and route	Cytokine analysis post immunization and post challenge by Bioplex
Control	PBS (i.n.), 2 doses	CO92-*lux* (100 LD_{50}), i.n.	Pro-inflammatory, Th1, Th2, and chemokines
Vaccinated	rAd5-YFV (i.n.), 2 doses		
Vaccinated	rAd5-LcrV (i.n.), 2 doses		

b

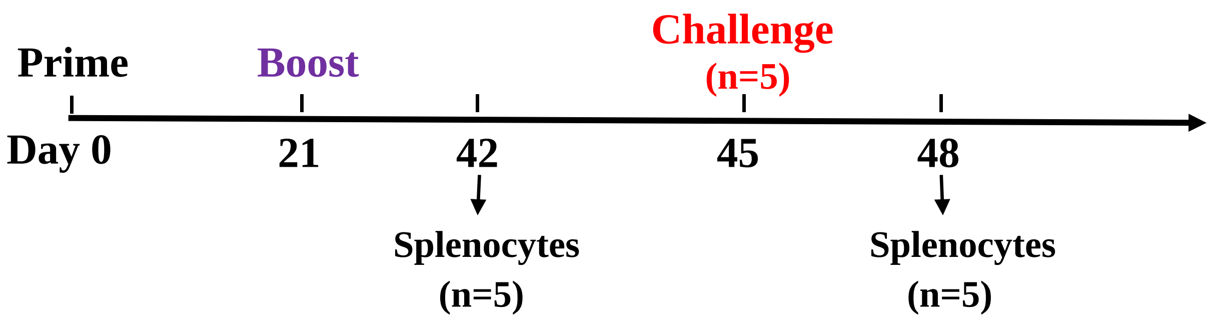

Supplementary Figure 2B.

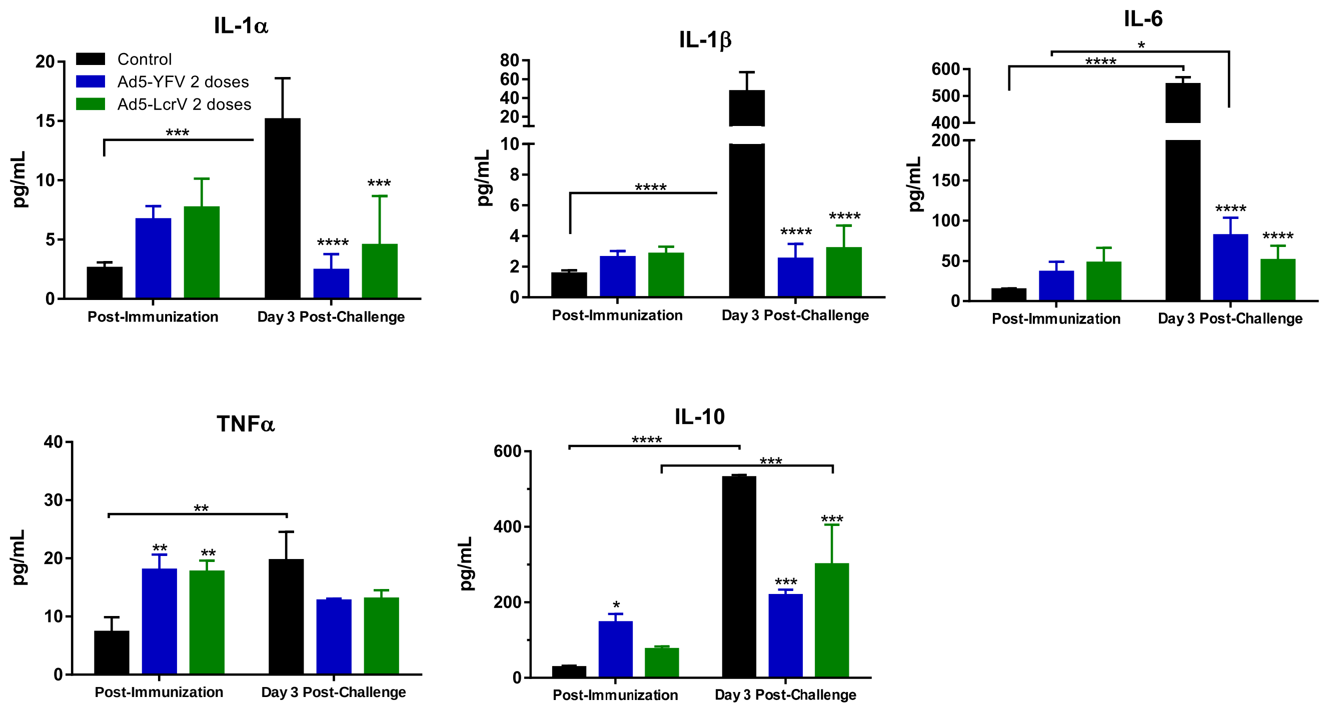

Supplementary Figure 2C.

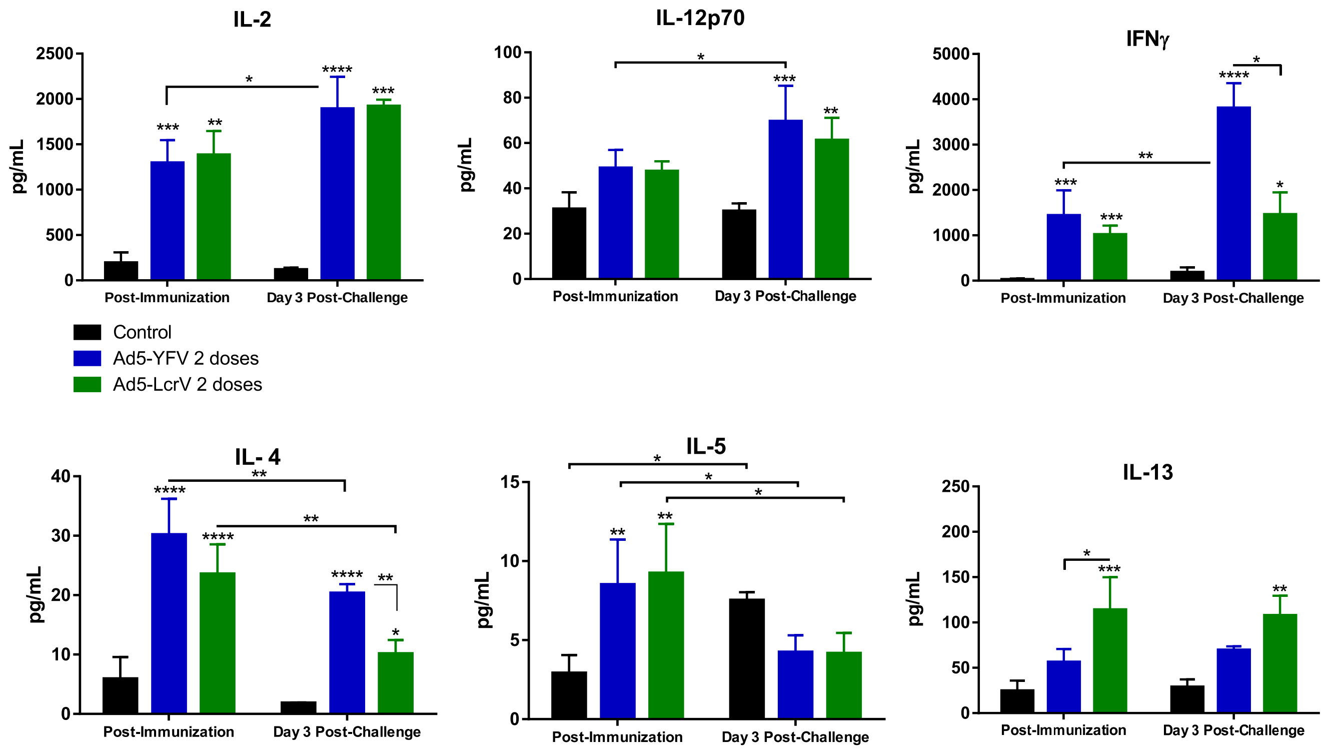

Supplementary Figure 2D.

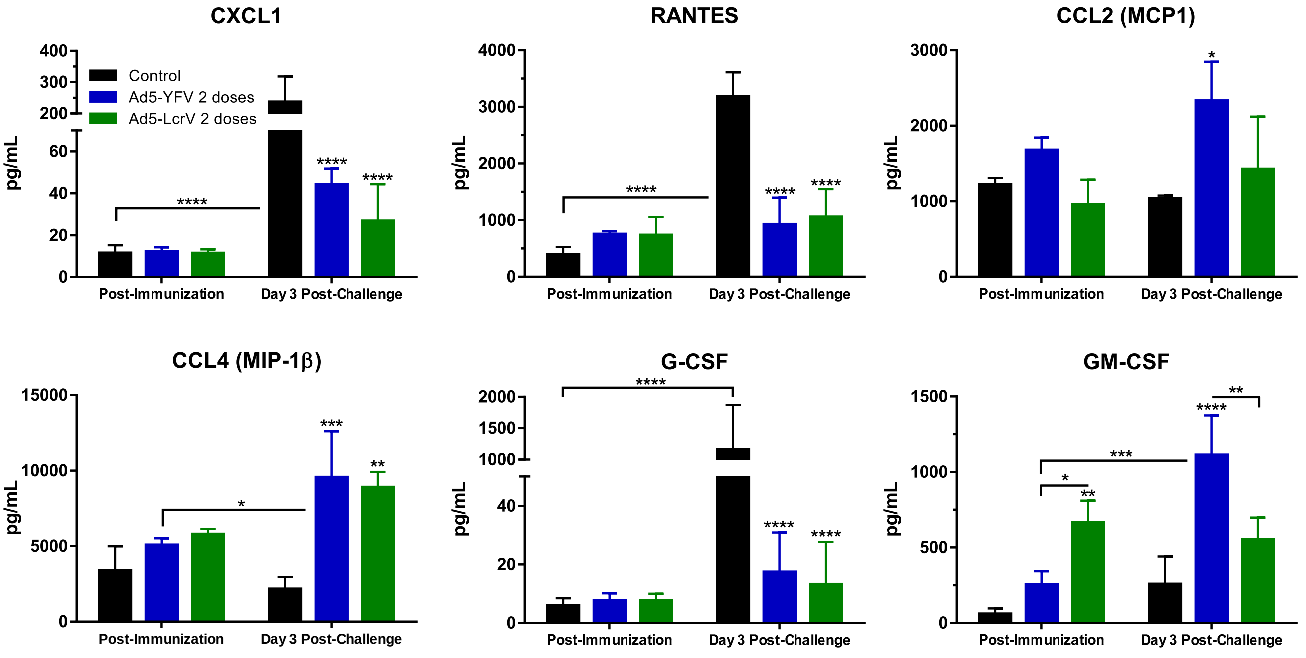

Supplementary Figure 2.Splenocyte cytokine and chemokine profiles in mice immunized with either rAd5-YFV or rAd5-LcrV vaccines. Mice (n=10/group) were immunized (i.n.) twice 21 days apart with $1.2x10^{10}$ v.p. of rAd5-YFV or rAd5-LcrV vaccines, with animals receiving PBS served as controls (**A-a**). Twenty-one days after the second vaccination dose or on day 3 post-challenge, spleens were harvested from mice (n=5/group/time point) and stimulated with purified F1-V (100 μg/mL) for 3 days (**A-b**). The cytokines in the culture supernatants were analyzed by using Bioplex-23 assay and expressed as the arithmetic means ± standard deviations. The proinflammatory and anti-proinflammatory cytokines were listed in (**B**), while the Th1 and Th2 cytokines were displayed in (**C**), and the chemokines as well as colony stimulating factors are presented in (**D**). P values were calculated using a two-way ANOVA with Tukey post hoc test to compare multiple time-points or One-way ANOVA with Tukey post hoc test to compare groups within the same time-point. Asterisks above columns represent comparison to the control group, while horizontal bars represent differences between test groups. *P<0.05, **P<0.01, ***P<0.001, **** P<0.0001. Two biological replicates were performed, and data plotted.

Supplementary Figure 3.

a

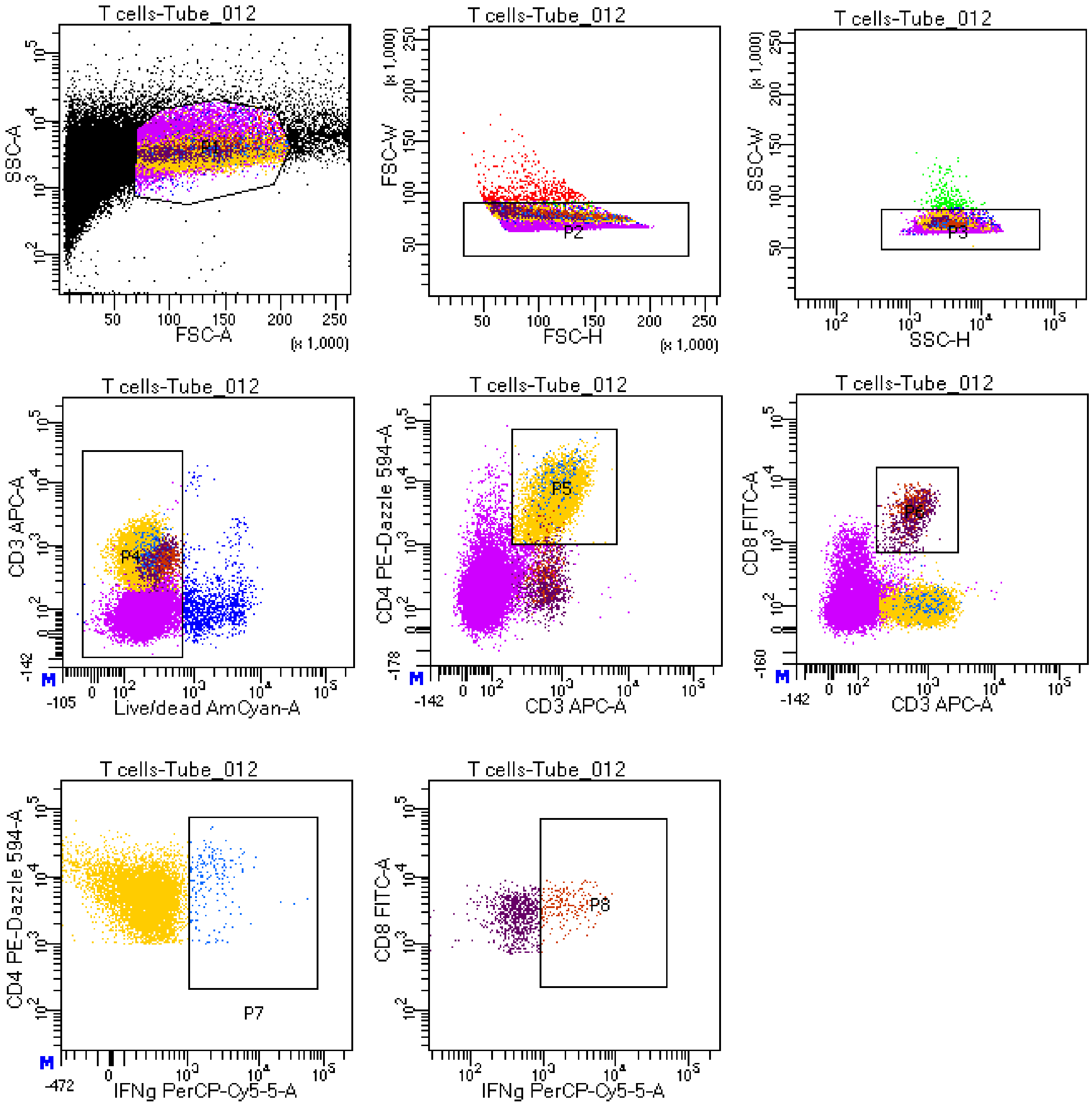

Tube: Tube_012

Population	#Events	%Parent	%Total
All Events	75,025	####	100.0
P1	24,896	33.2	33.2
P2	24,307	97.6	32.4
P3	24,049	98.9	32.1
P4	22,855	95.0	30.5
P5	7,276	31.8	9.7
P7	191	2.6	0.3
P6	1,051	4.6	1.4
P8	210	20.0	0.3

Supplementary Figure 3.

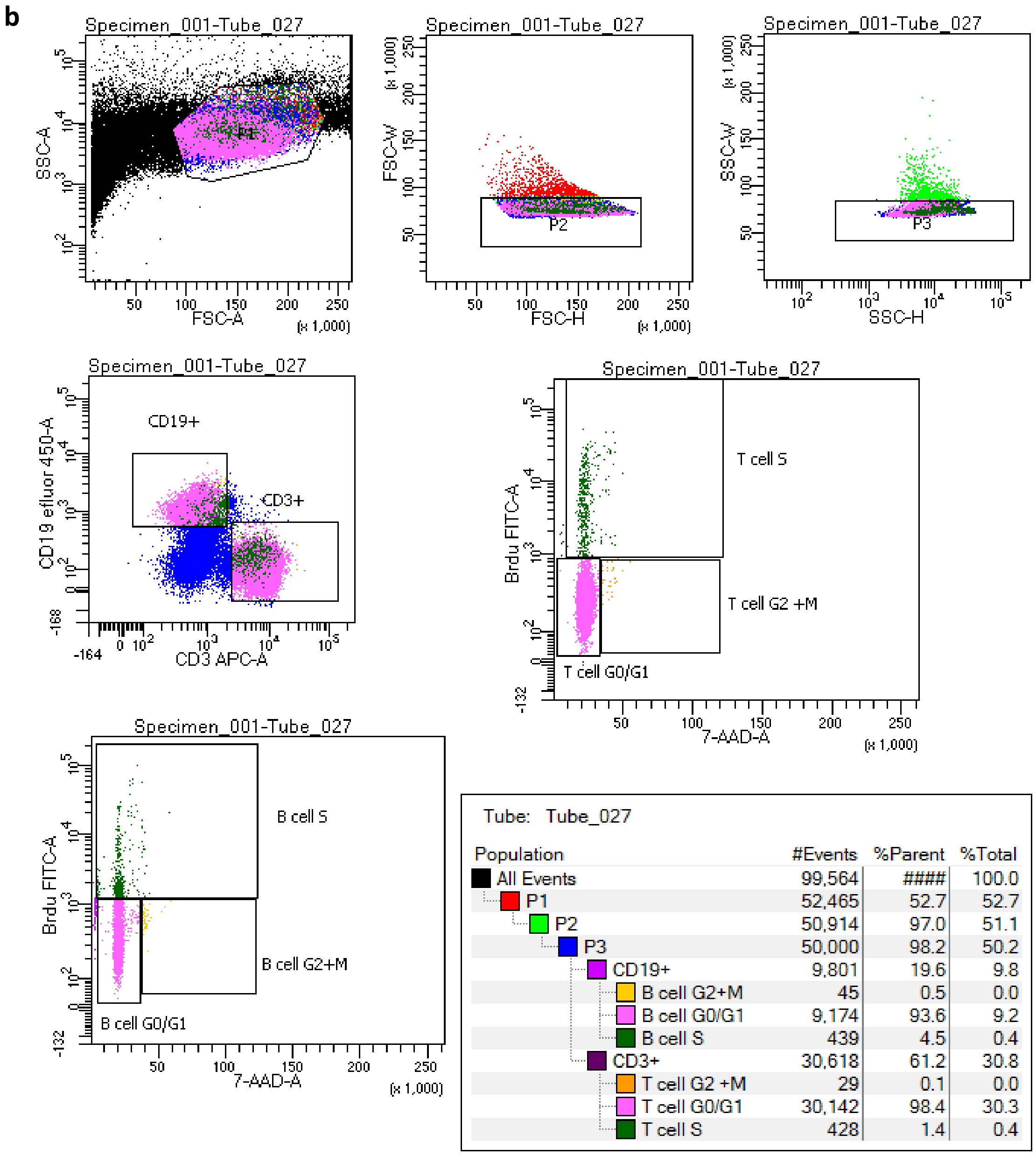

Tube: Tube_027

Population	#Events	%Parent	%Total
All Events	99,564	####	100.0
P1	52,465	52.7	52.7
P2	50,914	97.0	51.1
P3	50,000	98.2	50.2
CD19+	9,801	19.6	9.8
B cell G2+M	45	0.5	0.0
B cell G0/G1	9,174	93.6	9.2
B cell S	439	4.5	0.4
CD3+	30,618	61.2	30.8
T cell G2 +M	29	0.1	0.0
T cell G0/G1	30,142	98.4	30.3
T cell S	428	1.4	0.4

Supplementary Figure 3. Flow cytometry gating strategies. Lymphocytes were identified based on forward scatter (FSC) and side scatter (SSC) areas (FSC-A & SSC-A, respectively). Next, non-singlet cells were excluded based on FSC height (FSC-H) and width (FSC-W) characteristics as well as SSC height (SSC-H) and width (SSC-W) characteristics. In panel (**a**), dead cells were excluded based on uptake of cell-viability dye. T-cells (**a&b**) and B-cells (**b**) were identified by expression of CD3 (T-cells) and CD19 (B-cells). In panel (**a**), T-cells were further gated to identify $CD4^+$ or $CD8^+$ followed by IFNγ gating. In panel (**b**), T- and B- cells were gated on BrdU incorporation and 7-AAD staining to identify dividing cells that were in S phase of the cell cycle.
