# Supplementary material for: A new generation needle- and adjuvant-free trivalent plague vaccine utilizing adenovirus-5 nanoparticle platform
Source: NPJ Vaccines. 2021 Jan 29;6:21. doi: 10.1038/s41541-020-00275-3 (PMC7846801; doi:10.1038/s41541-020-00275-3)
Supplement: Supplementary file 2 — Reporting Summary [file 41541_2020_275_MOESM2_ESM.pdf]

nature research

Corresponding author(s): Ashok K. Chopra
Jian Sha

Last updated by author(s): Oct 23, 2020

Reporting Summary

Nature Research wishes to improve the reproducibility of the work that we publish. This form provides structure for consistency and transparency in reporting. For further information on Nature Research policies, see our Editorial Policies and the Editorial Policy Checklist.

Statistics

For all statistical analyses, confirm that the following items are present in the figure legend, table legend, main text, or Methods section.

n/a	Confirmed	
☐	☒	The exact sample size (*n*) for each experimental group/condition, given as a discrete number and unit of measurement
☐	☒	A statement on whether measurements were taken from distinct samples or whether the same sample was measured repeatedly
☐	☒	The statistical test(s) used AND whether they are one- or two-sided<br>*Only common tests should be described solely by name; describe more complex techniques in the Methods section.*
☒	☐	A description of all covariates tested
☐	☒	A description of any assumptions or corrections, such as tests of normality and adjustment for multiple comparisons
☐	☒	A full description of the statistical parameters including central tendency (e.g. means) or other basic estimates (e.g. regression coefficient) AND variation (e.g. standard deviation) or associated estimates of uncertainty (e.g. confidence intervals)
☐	☒	For null hypothesis testing, the test statistic (e.g. *F*, *t*, *r*) with confidence intervals, effect sizes, degrees of freedom and *P* value noted<br>*Give P values as exact values whenever suitable.*
☒	☐	For Bayesian analysis, information on the choice of priors and Markov chain Monte Carlo settings
☒	☐	For hierarchical and complex designs, identification of the appropriate level for tests and full reporting of outcomes
☒	☐	Estimates of effect sizes (e.g. Cohen's *d*, Pearson's *r*), indicating how they were calculated

Our web collection on statistics for biologists contains articles on many of the points above.

Software and code

Policy information about availability of computer code

Data collection	BD FACSDiva 8.0.1 software was used for flow cytometry data collection. For cytokine analysis, Bioplex 200 System software was used. For ELISA, SOFTmax PRO V.3.0 was used to read the plates. For IVIS imaging, IVIS 200 bioluminescent and fluorescence whole-body imaging workstation was used.
Data analysis	Graphpad Prism 6 was used to graph and analyze all data. BD FACSDiva 8.0.1 software was used to analyze flow cytometry data.

For manuscripts utilizing custom algorithms or software that are central to the research but not yet described in published literature, software must be made available to editors and reviewers. We strongly encourage code deposition in a community repository (e.g. GitHub). See the Nature Research guidelines for submitting code & software for further information.

Data

Policy information about availability of data

All manuscripts must include a data availability statement. This statement should provide the following information, where applicable:

- Accession codes, unique identifiers, or web links for publicly available datasets
- A list of figures that have associated raw data
- A description of any restrictions on data availability

All data that this study is based upon are available from the corresponding authors upon request.

Field-specific reporting

Please select the one below that is the best fit for your research. If you are not sure, read the appropriate sections before making your selection.

☒ Life sciences ☐ Behavioural & social sciences ☐ Ecological, evolutionary & environmental sciences

For a reference copy of the document with all sections, see nature.com/documents/nr-reporting-summary-flat.pdf

Life sciences study design

All studies must disclose on these points even when the disclosure is negative.

Sample size	For mice, our number of animals used per group will range from 6-10, which provides sound statistical significance. For example, if there are 10 animals per group, we will be 85% confident to statistically detect a 50% or larger drop in the read-out between groups at the 5% significance level. With 6 animals per group, we will be 72% confident to statistically detect a 50% or larger drop in the read-out.
Data exclusions	No data was excluded.
Replication	For animal studies, 2 biological replicates were performed.
Randomization	Mice were randomly assigned to cages by animal resources staff with no knowledge of the study design.
Blinding	Study was not blinded.

Reporting for specific materials, systems and methods

We require information from authors about some types of materials, experimental systems and methods used in many studies. Here, indicate whether each material, system or method listed is relevant to your study. If you are not sure if a list item applies to your research, read the appropriate section before selecting a response.

Materials & experimental systems

n/a	Involved in the study	
☐	☒	Antibodies
☒	☐	Eukaryotic cell lines
☒	☐	Palaeontology and archaeology
☐	☒	Animals and other organisms
☒	☐	Human research participants
☒	☐	Clinical data
☒	☐	Dual use research of concern

Methods

n/a	Involved in the study	
☒	☐	ChIP-seq
☐	☒	Flow cytometry
☒	☐	MRI-based neuroimaging

Antibodies

Antibodies used	Anti-CD3-APC,Thermofisher Scientific Cat: 17-0031-83<br>Anti-CD4-PE-Dazzle594 Biolegend Cat: 100566<br>Anti-CD8-FITC Biolegend Cat: 140403<br>Anti-IFNγ-PerCP/Cyanine5.5 Biolegend Cat: 505822<br>Anti-CD19-eFluor450 Thermofisher Scientific Cat: 48-0193-82<br>Anti-CD16/32 BioLegend Cat: 101302<br>FITC BrdU Flow Kit BD Bioscience Cat: 559619<br>Goat Anti-Mouse IgG, Human ads-HRP Cat:1030-05<br>Goat Anti-Mouse IgG1, Human ads-HRP Cat: 1070-05<br>Goat Anti-Mouse IgG2a, Human ads-HRP Cat: 1080-05<br>Goat Anti-Mouse IgA-HRP, Cat: 1040-05
Validation	We have provided examples of the gating strategy for each antibody used in supplemental figure 3. All antibodies have been validated for use in either flow cytometry or ELISA applications as described on the manufacturers website which can be found by looking up the Cat numbers provided above.

Animals and other organisms

Policy information about studies involving animals; ARRIVE guidelines recommended for reporting animal research

Laboratory animals	Six-to-eight-week old female Swiss-Webster mice were used in all studies.

Wild animals	N/A
Field-collected samples	N/A
Ethics oversight	The experiments were performed under an approved Institutional Animal Care and Use Committee protocol, UTMB, Galveston, Texas.

Note that full information on the approval of the study protocol must also be provided in the manuscript.

Flow Cytometry

Plots

Confirm that:

☒ The axis labels state the marker and fluorochrome used (e.g. CD4-FITC).

☒ The axis scales are clearly visible. Include numbers along axes only for bottom left plot of group (a 'group' is an analysis of identical markers).

☐ All plots are contour plots with outliers or pseudocolor plots.

☒ A numerical value for number of cells or percentage (with statistics) is provided.

Methodology

Sample preparation	Spleens were aseptically removed from the mice at designated time points. Spleens were homogenized and passed through a 70 µm cell strainer to obtain single cell suspension in RPMI 1640 cell culture medium.
Instrument	BD LSRFortessa Cell Analyzer
Software	BD FACSDiva 8.0.1
Cell population abundance	At least 50,000 events were analyzed for each sample. At least 90% of singlet cells were viable as determined by live-dead staining.
Gating strategy	Lymphocytes were identified based on forward scatter (FSC) and side scatter (SSC) areas (FSC-A & SSC-A, respectively). Next, non-singlet cells were excluded based on FSC height (FSC-H) and width (FSC-W) characteristics as well as SSC height (SSC-H) and width (SSC-W) characteristics. Dead cells were excluded based on uptake of cell-viability dye. T-cells and B-cells were identified by expression of CD3 (T-cells) and CD19 (B-cells). T-cells were further gated to identify CD4+ or CD8+ followed by IFNγ gating. For BrdU analysis, T- and B-cells were gated on BrdU incorporation and 7-AAD staining to identify dividing cells that were in S phase of the cell cycle.

☒ Tick this box to confirm that a figure exemplifying the gating strategy is provided in the Supplementary Information.
